# Digit Position and Forces Covary during Anticipatory Control of Whole-Hand Manipulation

**DOI:** 10.3389/fnhum.2016.00461

**Published:** 2016-09-15

**Authors:** Michelle Marneweck, Trevor Lee-Miller, Marco Santello, Andrew M. Gordon

**Affiliations:** ^1^Department of Psychological and Brain Sciences, University of California, Santa BarbaraSanta Barbara, CA, USA; ^2^Department of Biobehavioral Sciences, Teachers College, Columbia UniversityNew York, NY, USA; ^3^School of Biological and Health Systems Engineering, Arizona State UniversityTempe, AZ, USA

**Keywords:** grasp control, anticipatory planning, whole hand manipulation, object manipulation, digit position, load force, kinematics, kinetics

## Abstract

Theoretical perspectives on anticipatory planning of object manipulation have traditionally been informed by studies that have investigated kinematics (hand shaping and digit position) and kinetics (forces) in isolation. This poses limitations on our understanding of the integration of such domains, which have recently been shown to be strongly interdependent. Specifically, recent studies revealed strong covariation of digit position and load force during the loading phase of two-digit grasping. Here, we determined whether such digit force-position covariation is a general feature of grasping. We investigated the coordination of digit position and forces during five-digit whole-hand manipulation of an object with a variable mass distribution. Subjects were instructed to prevent object roll during the lift. As found in precision grasping, there was strong trial-to-trial covariation of digit position and force. This suggests that the natural variation of digit position that is compensated for by trial-to-trial variation in digit forces is a fundamental feature of grasp control, and not only specific to precision grasp. However, a main difference with precision grasping was that modulation of digit position to the object’s mass distribution was driven predominantly by the thumb, with little to no modulation of finger position. Modulation of thumb position rather than fingers is likely due to its greater range of motion and therefore adaptability to object properties. Our results underscore the flexibility of the central nervous system in implementing a range of solutions along the digit force-to-position continuum for dexterous manipulation.

## Introduction

Successful object manipulation is thought to rely on the use of stored internal representations of an object’s properties (Johansson and Westling, 1988; Gordon et al., 1991a,b, 1993). These representations are updated through anticipatory feedforward and feedback mechanisms (for a review Johansson and Flanagan, 2009). Most work informing the above theory studied either kinematics (hand shaping e.g., Jeannerod, 1984; Santello and Soechting, 1998; Santello et al., 2002; Ansuini et al., 2006; and digit positioning e.g., Cohen and Rosenbaum, 2004; Lukos et al., 2007) or kinetics (fingertip forces e.g., Westling and Johansson, 1987; Salimi et al., 2000, 2003; Reilmann et al., 2001; Pataky et al., 2004; Crajé et al., 2013). Studying these interdependent domains in isolation limits our understanding of their integration for planning and execution of object manipulation. For example, most studies on grasp kinetics focused how subjects modulate digit forces to object properties when grasping an object at fixed contact points. This task allows creating an internal representation of the forces, but only at those fixed contact points. Fu et al. (2010) addressed this limitation by studying planning of forces with unconstrained digit positions during two-digit object manipulation. The mass distribution was centered or off-centered and the task goal was to minimize object roll at lift onset. Visual cues of object properties were not salient (the object was symmetrically shaped but asymmetrical in mass distribution). Thus, internal representations formed during earlier experiences of the object were to be used to anticipatorily modulate position and forces. With unconstrained digit positions, subjects modulated *both* digit position and load forces to the object’s center of mass (CoM), e.g., higher load forces and digit positioning on the heavier object side. They found strong correlations between the vertical distance of thumb and index fingertip and forces on a trial-by-trial basis. Importantly, the compensatory moment (M*com*) countering the external torque of the CoM was statistically indistinguishable between the “unconstrained” and “constrained” grasp groups. The authors argued that modulating digit forces in response to digit position minimized M*com* variability. Digit position *and* force modulation has been replicated in other two-digit manipulation studies (Zhang et al., 2010; Fu and Santello, 2014; Marneweck et al., 2015). Thus, constraining digit position, like most previous work, prevents fundamental features of dexterous grasp control: (1) modulating digit position to object properties and task demands; and (2) modulating digit forces to compensate for trial-to-trial digit position variability.

Whether these phenomena of digit position modulation and successive force modulation are a general feature of grasp control, and not specific to precision grasp control, is unknown. For example, in another commonly employed grasp type, whole-hand grasping, four-finger positioning might not be modulated to the same extent as the index finger during two-digit (precision) grasping, given the former’s constraints to change individual finger position relative to the thumb during whole-hand grasping (Santello et al., 2013). In precision grasping, it was proposed that the functional role of modulating position was minimizing force and effort (given its link with lower grip force; Fu et al., 2010). In whole-hand grasping, the availability of more digits might weaken the need to implement a criterion of grip force minimization since they can simply alter the force distribution between digits (e.g., change force sharing patterns). Thus, digit position and forces during whole-hand grasping might not be modulated to the same extent as that found during precision grasping. Reports of differential neural circuits between precision and whole-hand grasping (e.g., Begliomini et al., 2007) further supports the possibility for differential behavioral idiosyncrasies between such grip types, such as how force and position is utilized during anticipatory planning of object manipulation.

Here, we determined digit position and forces modulation during anticipatory control of learned whole-hand object manipulation (object roll minimization) of a box with a centered and off-centered CoM, respectively. To achieve the task goal of minimizing object roll at lift onset, by matching the expected CoM location (and thus countering the external torque), digit position and force modulation must be anticipatory (in this case, based on prior lifting experience with the object), because no feedback about the actual CoM location is available until after lift onset. First, we hypothesized that subjects would modulate digit position and load force to the object’s CoM prior to lift onset (i.e., before sensory feedback signaling mass and its distribution becomes available). However, we expected less modulation of position by the four fingers than the thumb, given their aforementioned biomechanical constraints. Second, we hypothesized a strong trial-to-trial covariation in position and force.

## Materials and Methods

### Subjects

Twelve healthy adult subjects (eight females, age in years: *Mean* = 26, *SD* = 4) with normal or corrected-to-normal vision took part in the experiment. Subjects were right-handed (Oldfield, 1971) and reported no upper limb orthopedic impairments (or any other issue that might affect grasp performance). All subjects gave written informed consent to the study prior to testing in accordance with the Declaration of Helsinki. The experimental protocols were submitted to, and approved, by the Institutional Review Board at Teachers College, Columbia University.

### Materials and Procedures

**Figure 1:**
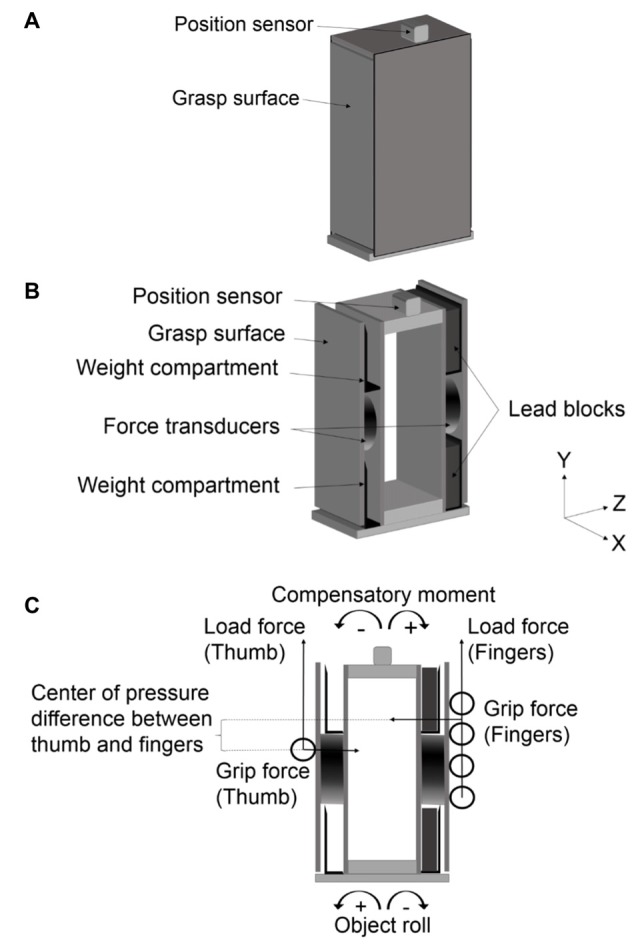
**A depiction of the custom-built rectangular box. (A)** The surfaces of the box as seen by the subject with the center of mass (CoM) concealed (thus the box always appeared symmetrical). **(B)** Grip surfaces of the box as attached to the force transducer, position sensor at the top of the box, and weight compartments that house the lead blocks above and below the force transducers to create an off-centered mass distribution. **(C)** Load force, center of pressure (COP), grip force and compensatory moment (M*com*) taken by the force transducers on the thumb side and on the four finger side, respectively and object roll as measured by the position sensor.

Subjects were asked to grasp and lift, using a whole-hand grip, a rectangular box with a concealed CoM that was centered or off-centered (on the left or right side of the box; Figure 1). The aim of the task was to prevent object roll.

The surfaces of the rectangular box (height: 16.5 cm; width: 8 cm; depth: 8.5 cm) concealed the position of the added mass (see below). Thus, when the CoM was centered, the object was symmetrical in appearance and in mass distribution. When the CoM was off-centered, the object was still symmetrical in appearance, but not in mass distribution (Figure 1A). To vary the mass distribution, two lead blocks (each with a height of 5 cm, width of 8 cm, depth of 1 cm, and mass of 370 g) were placed on the left, center, or right side of the box (Figure 1B). The external torque created by a left or right mass distribution was ±21.74 Ncm.

The two lateral grip surfaces of the box were made of carbon fiber (height: 15.2 cm; width: 7.6 cm; thickness: 3 mm). The carbon fiber sheet was covered with a thin balsawood sheet (thickness: 2 mm) to cover the screws that attached the carbon fiber sheet to the force transducer. Sandpaper was affixed to the balsawood to increase the friction between the digits and the object’s contact surface. The front, back, and top side of the box were detachable as a unit from the grip surface sides to easily shift the CoM (Figure 1B).

A force/torque transducer (Mini 40, ATI Industrial Automation, NC, USA) was affixed to each of the grip surfaces. The transducers measured load force, grip force and moments exerted by the digits with resolutions of 0.01 N, 0.02 N and 0.0125 Ncm, respectively. Note that the force/torque transducer on the finger side of the grip device measured the total grip and load forces exerted by all fingers, as well as the net moment of all fingers relative to the center of the sensor. An electromagnetic position-angle sensor (Polhemus Fastrack; angular resolution: 0.025°; displacement resolution: 0.0005 cm) was attached at the top of the box to measure object roll. The total mass of the box, with the force transducers, position-angle sensor, and lead blocks was 1270 g. Fingertip force data were sampled at 500 Hz and position data were sampled at 120 Hz using SC/Zoom (Umeå University, Sweden). Data collected were filtered using a second-order low pass Butterworth filter with a cut-off of 6 Hz, and the force/torque transducer data were synchronized by interpolating with position data offline.

A webcam was affixed to the edge of the table, 45 cm to the grasp surface of the box and in line with the center of the box, to record the position of the index, middle, ring, and little fingertips (resolution: 640 × 480; frame rate: 25 frames/s). This recording was done because individual position of the four fingertips could not be determined from the center of pressure (COP) recorded by the force transducer, which can only record the net COP of all four fingertips (see below). The thumb position was determined from the COP recorded by that transducer.

Subjects sat in front of a height-adjustable table facing the box with the right elbow flexed approximately 90° in the parasagittal plane. The right shoulder was aligned with the midpoint of the box. The right hand was placed palm facing down at a marked start location, which was 12 cm from the midpoint of the box. Following an auditory cue, subjects were instructed to reach from the marked start location, grasp the grip surfaces with the tips of the thumb and fingers of the hand, and lift the box at a self-selected speed to the height of an adjacent marker (10 cm). Following a second auditory cue, occurring 1.5 s after the vertical distance of the box exceeded 6 cm, subjects were instructed to replace both box and hand back to their respective start locations. Subjects were asked to minimize the roll of the box as best as they could. No instruction was given regarding fingertip position on the box.

There were three blocks of trials, each corresponding to the CoM on the left, center and right side (with the block order following a Latin Square sequence across subjects). When the CoM was on the left, subjects were to produce a supination moment to counter the external torque of the mass. When the CoM was on the right, subjects were to produce a pronation moment to counter the external torque caused by the added mass. For each block, there were five practice trials and 20 test trials with a 5-s inter-stimulus interval between trials (recorded from the second auditory cue). Practice trials were given to ensure correct execution of the task during the 20 test trials, which was the main focus of analyses. The number of practice trials was chosen based on previous studies with similar tasks that showed that subjects can learn an object roll minimization task within three trials (e.g., Fu et al., 2010).

### Data Analyses

We quantified peak roll (in degrees) on the frontal plane of the box occurring after lift onset. Lift onset was defined as the time at which the vertical position of the object (as measured from the table surface) exceeded 1 mm and continued to increase thereafter. Subjects at times would exceed this 1-mm position criterion by means of a movement in a roll or pitch direction (with some part of the box not fully lifted from the table). However, we chose this stringent criterion for defining object lift onset to avoid as best possible any influence of feedback signaling mass and distribution on our measures, which were primarily focused on anticipatory control of digit position and forces. Positive and negative values denote rolls in the direction of the thumb and fingers, respectively.

We recorded digit load force at lift onset, the vertical force component parallel to the grip surface, exerted on the thumb and on the four fingers, respectively. We computed the difference between these load forces (ΔF*y*), such that a zero value denotes symmetrical load forces exerted by the thumb and four fingers. A positive ΔF*y* value denotes that the thumb exerted more load force than the four fingers combined, whereas a negative ΔF*y* value denotes that the four fingers combined exerted more load force than the thumb.

The COP was computed for the thumb and the four fingers at lift onset. COP is defined as the vertical coordinate of the point of resultant force relative to the center of the force transducer:

(1) $$COP\;=\;[Tx\;-\;(Fy^{*}w)]/Fz],$$

where T*x* is the moment about the *x*-axis, F*y* is the load force, *w* is the distance between the surfaces of the force/torque transducer and the grip surface (0.5 cm), and F*z* is the mean grip force component perpendicular to the grip surface averaged across thumb and four fingers (Figure 1C). We took the difference between the COP of the thumb and the net COP of the four fingers (ΔCOP). A positive ΔCOP value denotes that the thumb COP was higher than the net COP of all fingers, whereas a negative ΔCOP value denotes that the net COP of the four fingers was higher than the thumb COP. Furthermore, the M*com* (Ncm) was computed using the formula:

(2) $$Mcom\;=\;[(\Delta Fy{)}^{*}d/2+Fz^{*}\Delta COP],$$

where *d* is the grip width (8 cm). Positive and negative M*com* values denote M*com* generated in the direction of the fingers and thumb, respectively.

As mentioned above, the COP of individual fingers could not be determined from the force and torque output of the force transducer (as was done for the position of the thumb). Thus, to determine the position of each fingertip, we used a webcam to record fingertip position on each test trial. Digit position data were extracted at two time points: at the first frame showing vertical motion of the box and at the frame 0.16 s before that. The vertical distance between the centroid of each fingernail and the center of the grip surface was measured using video-based movement analysis software (DartFish Pro Suite 9.0^TM^, Fribourg, Switzerland), using the height of the box as the environmental reference point. There was no statistically significant difference in the position of the four fingertips extracted at the two frames (*p*’s > 0.05), thus we used the frame 0.16 s before the object lift onset frame.

Our main focus was on examining the kinetics and kinematics of *successful* anticipatory control of whole-hand manipulation, and their possible relation. To do so, we used three-level one-way repeated measures analysis of variance (ANOVAs) to compare the following variables averaged across 20 trials per subject across the three CoM conditions: peak object roll, M*com*, F*z*, ΔF*y* and ΔCOP. Furthermore, we compared the extent to which digit position of the four fingers, as captured by our webcam data, varied across CoM conditions using a 4 × 3 repeated measures ANOVA (with *Fingers* and *CoM* as within-subject factors). For one subject, we removed one of the 20 trials (one CoM condition) because the force transducer was overloaded. For significant main effects, we performed Tukey *post hoc* tests. We report partial eta squared ($\eta_{p}^{2}$) as a measure of effect size. Finally, for each subject we calculated Pearson’s correlations between ΔF*y* and ΔCOP (20 values for each CoM condition). A mean correlation coefficient *r* was then calculated for each CoM condition using Fisher’s *r-z* transformation.

## Results

**Figure 2:**
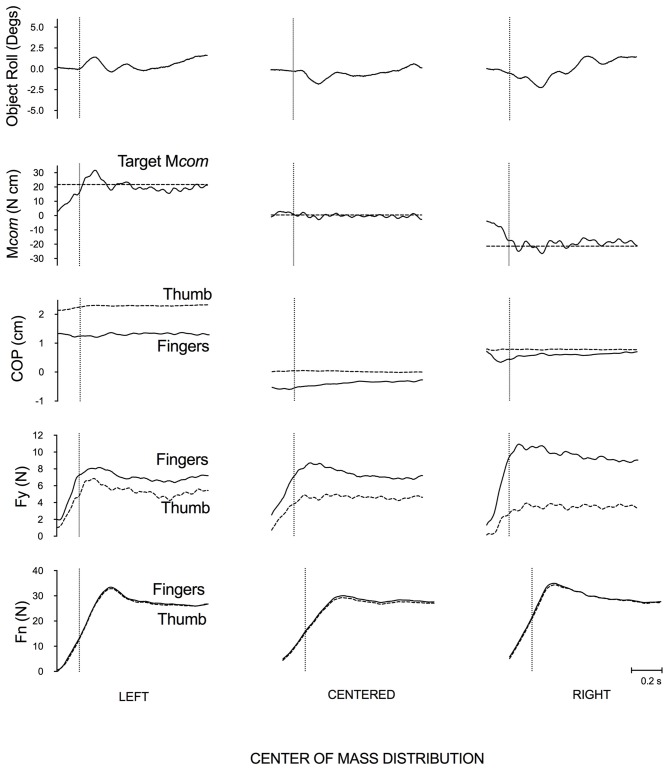
**Data from representative subject performing the task with the box for each CoM condition.** From top to bottom, data shown are object roll, the generated (solid line) and target (horizontal dotted line) M*com*, the COP of thumb (dotted line) and four fingers (solid line), load force (F*y*) and grip force (Fn) exerted by the thumb (dotted line) and the four fingers (solid line). Left, center, and right columns show trials when grasping and lifting the box with a left, centered and right mass distribution, respectively. The vertical dotted line in each figure denotes lift onset. Note that the target M*com* was not displayed as visual feedback to subjects.

Figure 2 shows data from a representative subject who grasped and lifted the box on a test trial with a left, right and centered mass distribution, respectively. When manipulating the box with a centered mass distribution, the subject did not need to generate a M*com* and therefore the box did not roll. When manipulating the box with an off-centered mass distribution, the subject had to generate an appropriate M*com* in the opposite direction of box’s CoM (e.g., clockwise/supination and counterclockwise/pronation when the mass was on the left and right side, respectively) to minimize object roll. Interestingly, for this subject the COP of the thumb was always higher than that of the net COP of the fingers, and the load force of the four fingers was always higher than that of the thumb, regardless of the box’s mass distribution.

**Figure 3:**
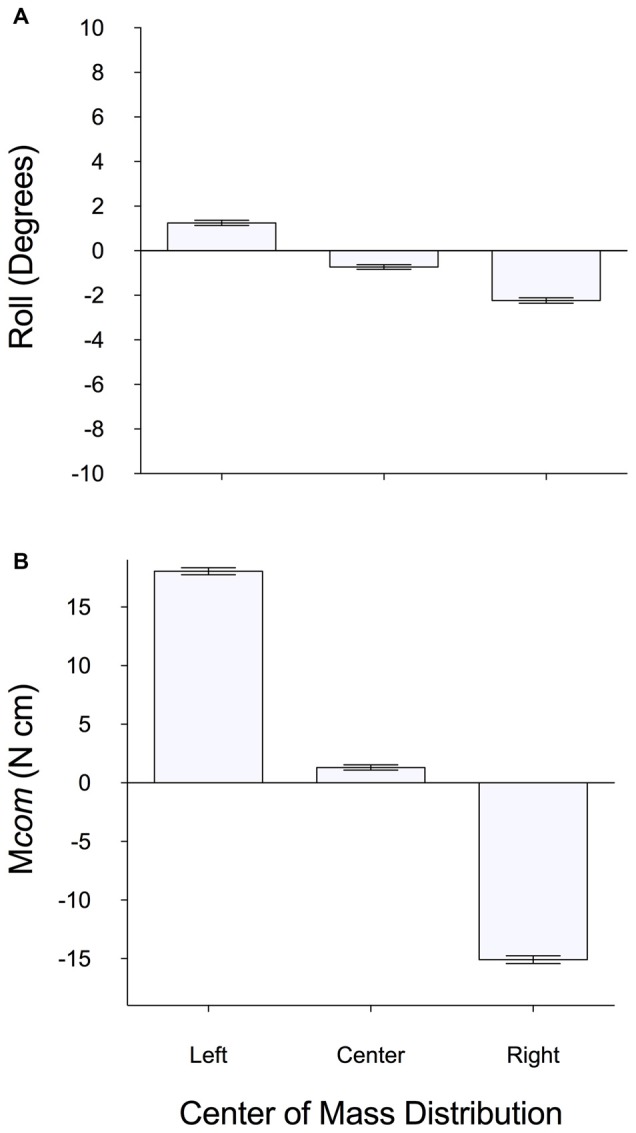
**Mean (±1 SE) of subjects’ test trials of (A) object roll and (B) M*com* when grasping and lifting a box with a left, centered and right mass distribution.** For roll, positive and negative values indicate roll towards left and right side of the box, respectively. For M*com*, positive and negative values indicate a M*com* generated away from the left and right side of the box, respectively.

The above results were also generally found across all subjects (Figure 3). We found significant main effects of *CoM* (left, right, centered) on M*com* (*F*_(2,22)_ = 354.70, *p* < 0.05, $\eta_{p}^{2}$ = 0.97) and object roll (*F*_(2,22)_ = 28.53, *p* < 0.05, $\eta_{p}^{2}$ = 0.72), and large effect sizes. Tukey *post hoc*s showed significant differences in M*com* and roll when comparing left, right and centered mass distributions.

**Figure 4:**
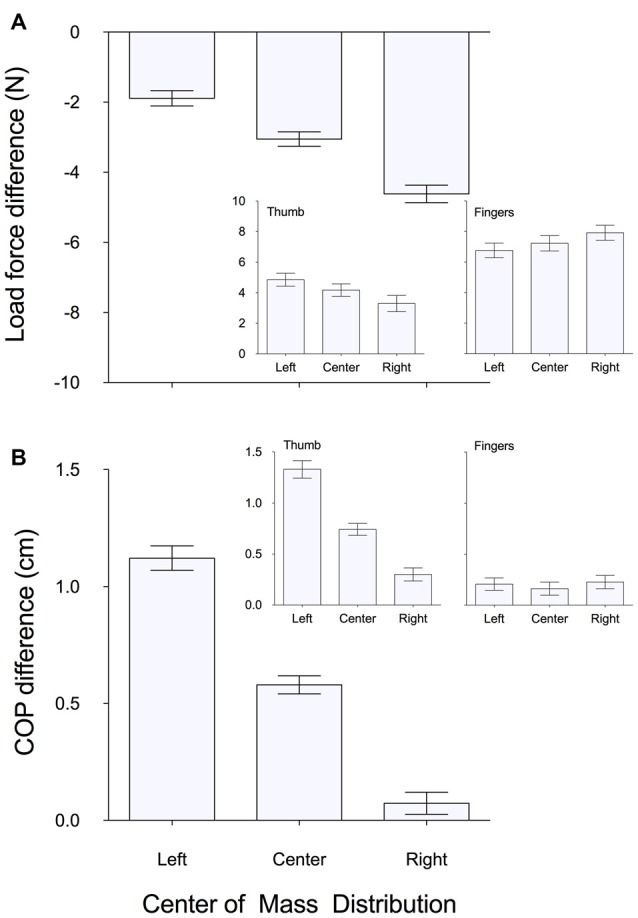
**Mean (±1 SE) of subjects’ test trials of (A) load force difference and (B) COP difference between the thumb and the four fingers when grasping and lifting a box with a left, centered and right mass distribution.** Positive values indicate more load force and higher COP exerted by the thumb than the four fingers. Negative values indicate more load force and higher COP exerted by the four fingers than the thumb. Inserts in panel **(A)** show load force by the thumb (left) and four fingers (right) for left, center and right mass distribution conditions. Inserts in panel **(B)** show COP by the thumb (left) and four fingers (right) for left, center and right mass distribution conditions.

Figure 4 shows that in all CoM conditions, subjects generally exerted higher load forces by the fingers than thumb. Nevertheless, there was a significant main effect of *CoM* (left, right, centered) on ΔF*y* (*F*_(2,22)_ = 5.09, *p* < 0.05, $\eta_{p}^{2}$ = 0.32), with Tukey *post hoc*s showing a significant difference between left and right CoM conditions. F*y* by the thumb and the net F*y* by the fingers were also each, respectively, significantly modulated based on the *CoM* (thumb: *F*_(2,22)_ = 5.44, *p* < 0.05, $\eta_{p}^{2}$ = 0.33; fingers: *F*_(2,22)_ = 3.54, *p* < 0.05, $\eta_{p}^{2}$ = 0.24) with Tukey *post hoc*s showing significant differences between left and right CoM conditions.

**Figure 5:**
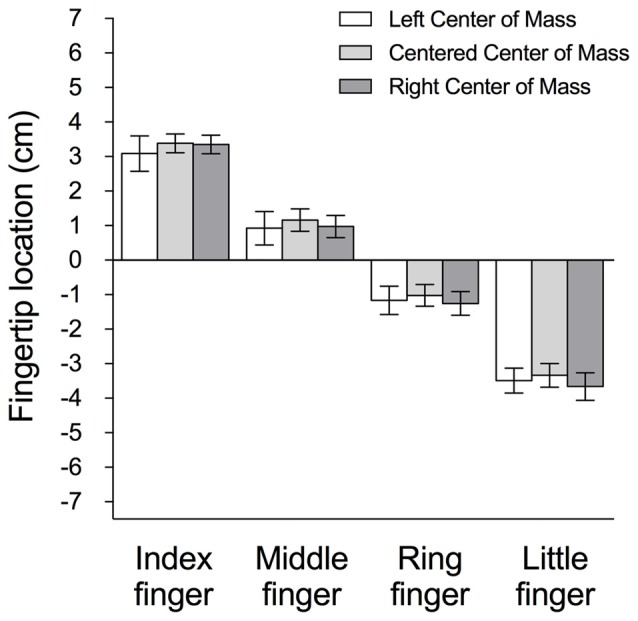
**Mean (±1 SE) of subjects’ test trials of digit position, measured as the distance from the tip of the index, middle, ring and little finger to the center of the force transducer, for the left (clear), centered (light gray) and right (dark gray) CoM conditions.** Digit position above and below the center of the force transducer is a positive and negative value, respectively.

Figure 4 also shows, consistent with the above data from a representative subject, that subjects generally tended to grasp the object with the thumb COP higher than that of the fingers in all CoM conditions. Nevertheless, we found a significant main effect of *CoM* (left, right, centered) on ΔCOP (*F*_(2,22)_ = 10.51, *p* < 0.05, $\eta_{p}^{2}$ = 0.49), and Tukey *post hoc*s showed a significant difference between the left and both right and centered CoM conditions. Interestingly, we observed a stronger COP modulation to object CoM in thumb COP than in the net COP of the fingers (Figure 4B). This was further quantified by a main effect of *CoM* on thumb COP (*F*_(2,22)_ = 4.79, *p* < 0.05, $\eta_{p}^{2}$ = 0.30) but not on fingers COP (*p* > 0.05). Tukey *post hoc*s showed a significant difference between thumb COP from left and right CoM conditions. Furthermore, the individual position of the four fingertips (as captured by the webcam) varied little across CoM conditions (Figure 5), with no main effect of *CoM* (*p* > 0.05) and no interaction between *Finger* and *CoM* (*p* > 0.05). Of note, there was no significant difference (*p* > 0.05) in mean grip force at lift onset of a box with a left, right, or centered mass distribution. There were also no significant effects of trial number on thumb COP (or any other measure; *p*’s > 0.05) in any of the CoM conditions. Together, these findings suggest that the modulation in ΔCOP to the object’s CoM was largely driven by changes in the thumb COP rather than the position of the four fingertips.

**Figure 6:**
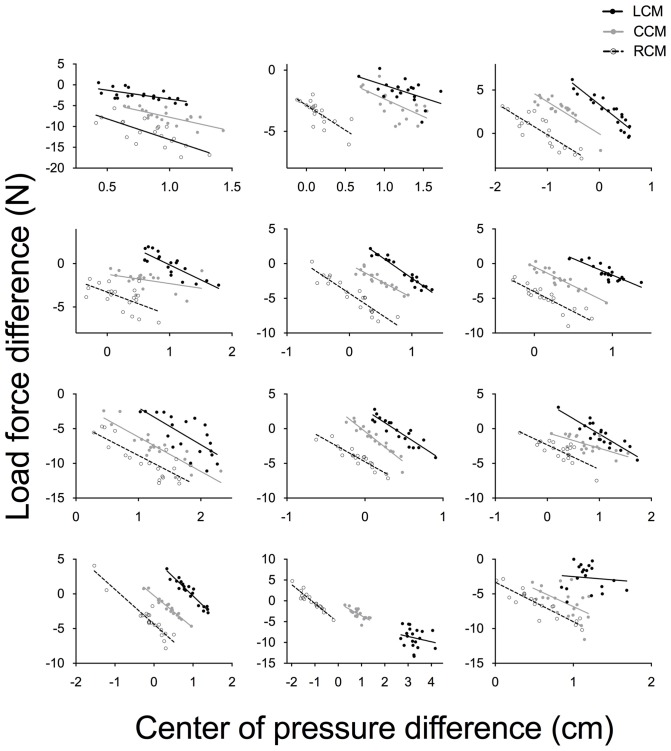
**Individual scatterplots for each of the 12 subjects showing the relationship between the measure of load force difference (between the thumb and the four fingers) and COP difference (between the thumb and the four fingers).** Data points of trials with the CoM of the box distributed on the left (LCM), center (CCM), and right (RCM) are represented by solid, gray and clear symbols, respectively. The best-fitting straight line for each of the three conditions is shown in each panel. Note that difference scales were used for different subjects for illustration purposes.

Our correlational analyses showed very strong, negative and significant correlations between ΔF*y* and ΔCOP in each condition (Figure 6; this figure also shows some exceptions to what was typically seen in load force difference and ΔCOP, e.g., see subject depicted in subfigure at top right, right panel, showing a positive load force difference and negative ΔCOP). The mean correlation coefficient *r* (calculated after Fisher’s *r-z* transformation) was 0.81 (95% CI: 0.57, 0.92), 0.86 (95% CI: 0.68, 0.94), and 0.86 (95% CI: 0.66, 0.94) for the left, centered and right mass distribution conditions, respectively. Altogether, these findings suggest that the strong covariation of both fingertip position (driven by the thumb) and load force contributed to successful whole-hand object manipulation.

## Discussion

This study measured the extent to which digit position and forces are modulated for anticipatory control of learned manipulation of objects using the whole hand. Our findings supported our first hypothesis that digit position and force are modulated based on object CoM, with digit position modulation being limited to thumb position, and little to no modulation of finger position. Our second hypothesis of strong trial-to-trial covariation between digit position and load force was also supported. To our knowledge, this study is the first to have measured digit force-to-position modulation and its covariation in a grip type other than a two-digit precision grip type. Our results suggest that digit force modulation to position—necessitated by the need to perform a given manipulation in a consistent fashion despite trial-to-trial variability in digit placement—is a fundamental and ecological feature of grasp control regardless of the number of fingers involved in the grasp.

### Digit Force-to-Position Modulation in Whole-Hand Grasping and Manipulation

In achieving the task goal of minimizing the roll of a box with an off-centered mass distribution, subjects could have utilized three possible solutions. First, and as has been shown in precision grasping (Fu et al., 2010; Zhang et al., 2010; Marneweck et al., 2015), subjects could have modulated *both* digit position and load forces, with higher digit positioning and larger forces on the heavier side of the object. Our results show that this solution was only partially implemented in our task (see third solution below). Second, subjects could have modulated forces *only* while using the same digit position regardless of object CoM. Studies on constrained grasping have shown that subjects can modulate digit forces at fixed digit placement when they are not given the option of modulating digit position (for a review, Zatsiorsky and Latash, 2008). In our unconstrained grasp scenario, however, subjects did modulate digit position *and* force. Third, subjects could have modulated digit position to the CoM mostly by the thumb and not the fingers, given the biomechanical limitations of all fingers to move relative to each other and the thumb in a whole-hand grasp. This third solution is consistent with our results, and provides the first description of unconstrained digit force modulation for whole-hand grasping and manipulation.

The greater modulation of the thumb position than fingers is likely due in part to the thumb’s greater range of motion and degree of independence. Specifically, the thumb can abduct to a greater degree than the fingers (70° vs. 30° in the index finger; Marzke, 1994; Jones and Lederman, 2006). Another unique feature of the thumb, due to the articulation of the thumb metacarpal and the trapezium, is that it can rotate 45° around its longitudinal axis. The greater modulation of the thumb position than the fingers is also likely due to biomechanical limitation of all fingers to move independently from each other and from the thumb. Finally, it might be worth considering whether the position of the four fingers was modulated less than that of the thumb because there was less room along the grasp surface for the fingers to be modulated. We considered this during the design of the vertical dimension of the grasp surface. The grasp surface height was set to 15 cm, based on how much digit position has been shown to vary in previous 2-digit grip studies (e.g., ~1.5–2 cm) utilizing similar experimental paradigms, and given that the maximum finger span (distance between index and little fingertip) of an average hand does not exceed 15 cm. Therefore, it is unlikely that the height of the grasp surface played a significant role in the limited position modulation of the fingers compared to the thumb.

### Digit Force and Position Covariation in Whole-Hand Grasping and Manipulation

Like that seen in precision grasping (Fu et al., 2010), the linear negative correlation between trial-to-trial distance between digit COP and load force difference was very strong. Thus, the coordination of COP and load force is indeed a critically important phenomenon for grasp manipulation, at least for two commonly employed grasp types, e.g., two-digit grasp (e.g., Fu et al., 2010) and whole-hand grasp (present work). Despite this trial-to-trial variability in forces and digit position (with variability in the latter seen mainly in the thumb), the M*com* required by our task goal (object roll minimization) was nevertheless attained. Our findings further support the explanation put forth previously (Fu et al., 2010) that subjects are able to compensate for trial-to-trial variability in digit positioning through anticipatory modulation of forces. Little to no modulation of finger positioning resulted in a net negative load force difference between the thumb and the fingers in all conditions (though of different magnitudes), indicating the fingers always exerted more load force to account for modulation of the thumb position. A previous study has reported this same pattern, albeit during the static phase of lifting an object with a centered CoM using a whole-hand grasp at constrained contacts (Reilmann et al., 2001). They suggested that the load force by the four fingers had to compensate for the negative/downward load force in the thumb. Similarly, subjects in the present study could have increased load forces in their fingers to compensate for less load force in the thumb. Furthermore, since we did not constrain digit positioning, subjects placed their thumb at a position that might have spared them from the need to modulate thumb load force. A limitation of our study was that we could not measure *individual* forces exerted by each of the four fingers. Specifically, it is possible that the four fingers used an additional strategy to minimize object roll by redistributing forces among the four fingers and altering the force sharing pattern as shown in other studies of whole-hand object manipulation at constrained contacts (e.g., Santello and Soechting, 2000; Rearick and Santello, 2002; Zatsiorsky et al., 2003; Shim et al., 2005). Thus, the ability to redistribute forces among the four individual fingers might reduce the need or outweigh any additional gain of modulating digit positions.

### Theoretical Considerations for Anticipatory Control of Object Manipulation

We currently understand anticipatory control of object manipulation to rely on visual feedback and on sensorimotor memories from prior interactions with the same or similar object (for a review, see Johansson and Flanagan, 2009). Specifically, anticipatory digit force control is based on comparing actual sensory consequences, obtained from feedback mechanisms, with sensory consequences that we expect based on upcoming motor actions, derived from feedforward mechanisms, at a series of crucial time events. In the event of a mismatch between the actual and planned sensory consequences, modulation of forces would be triggered, and the associated sensorimotor memory might be updated.

Most previous work informing this theoretical perspective has been studied using either kinematics (measuring hand shaping e.g., Ansuini et al., 2006 and digit positioning e.g., Cohen and Rosenbaum, 2004) or kinetics (measuring fingertip forces e.g., Bursztyn and Flanagan, 2008). Our work and that from a previous study (Fu et al., 2010) suggest an update to this theoretical perspective, with a specific inclusion of the evidently strong integration of kinematical and kinetic variables during the loading phase of motor control. That a stable M*com* is still reached, despite trial-to-trial variation in digit positioning supports the system’s ability to sense digit position *during* load phase and integrating this information very swiftly to modulate load forces accordingly prior to object lift onset. What might enable this rapid sensing of digit positioning prior to load force modulation before the object is lifted? Our findings of trial-to-trial variation in digit position (of the thumb) and forces suggest anticipatory modulation of forces would not only depend on sensorimotor memory of forces and digit positioning from previous grasping experience, but also on feedback from the actual positioning of the fingertips. Fu et al. (2010) proposed that such feedback of digit position is likely acquired before digit contact, and between digit contact and lift onset, via visual, tactile, and proprioceptive inputs. Subsequently, a comparison is made between the expected and actual feedback of digit position, with a mismatch resulting in a change in the planned digit forces. This means that the forces originally planned before contact (based on the anticipated CoM location from prior lifts) would require online monitoring following contact, and occurring possibly through an integration of proprioception, tactile and visual input, and correcting to compensate for positioning variation. The mechanisms that allow such swift integration of digit position variability, and subsequent load force adjustment, prior to lift onset, remains to be elucidated. Nevertheless, a study by Davare et al. (2007) gives neurophysiological support for the proposition that forces are modulated in response to digit positioning during anticipatory planning of object manipulation. Specifically, they showed that repetitive transcranial magnetic stimulation (rTMS) applied over anterior intraparietal area (AIP) 270–220 ms before object contact disrupted digit positioning, whereas rTMS at a later time point, 170–120 ms before object contact, disrupted digit forces. Although our study designs varied (e.g., their design had no specific metric of task correctness such as object roll minimization in our design), both our results point to force modulation being planned in response to digit positioning during anticipatory planning of object manipulation.

Reminiscent of the classic concept of “motor equivalence” (Lashley, 1930; Cole and Abbs, 1986), the ability to generate an appropriate M*com* despite trial-to-trial variation in digit position and force suggests the presence of a higher order motor plan or neural representation that codes the task goal independent of the variety of ways in which it can be reached. Our findings suggest that the natural variation of digit position, for which digit forces must compensate on trial-to-trial fashion, is a fundamental feature of grasp control regardless of the number of fingers involved in the grasp. These findings support humans’ ability to monitor online whether planned and actual digit position coincide, and to correct for a possible mismatch, by modulating load force. This phenomenon is critically important to ensure that the desired force and/or torque magnitude is attained by the time manipulation can be initiated.

## Author Contributions

Designed the experiment: MM, TL-M, MS, AMG. Performed the experiment: MM, TL-M. Analyzed and interpreted the data: MM, TL-M, MS, AMG. Wrote and edited the manuscript: MM, TL-M, MS, AMG. Approved the final version of the manuscript: MM, TL-M, MS, AMG. Agreed to be accountable for all aspects of the work: MM, TL-M, MS, AMG.

## Funding

This research was supported by a Collaborative Research Grant BCS-1455866 (MS) and BCS-1455865 (AMG) from the National Science Foundation (NSF; http://www.nsf.gov).

## Conflict of Interest Statement

The authors declare that the research was conducted in the absence of any commercial or financial relationships that could be construed as a potential conflict of interest.
